# Suppression of Sensitivity to Drugs and Antibiotics by High External Cation Concentrations in Fission Yeast

**DOI:** 10.1371/journal.pone.0119297

**Published:** 2015-03-20

**Authors:** John P. Alao, Andrea M. Weber, Aidin Shabro, Per Sunnerhagen

**Affiliations:** Department of Chemistry and Molecular Biology, University of Gothenburg, Box 462, SE-405 30, Göteborg, Sweden; University of Cambridge, UNITED KINGDOM

## Abstract

**Background:**

Potassium ion homeostasis plays an important role in regulating membrane potential and therefore resistance to cations, antibiotics and chemotherapeutic agents in *Schizosaccharomyces pombe* and other yeasts. However, the precise relationship between drug resistance in *S*. *pombe* and external potassium concentrations (particularly in its natural habitats) remains unclear. *S*. *pombe* can tolerate a wide range of external potassium concentrations which in turn affect plasma membrane polarization. We thus hypothesized that high external potassium concentrations suppress the sensitivity of this yeast to various drugs.

**Methods:**

We have investigated the effect of external KCl concentrations on the sensitivity of *S*. *pombe* cells to a wide range of antibiotics, antimicrobial agents and chemotherapeutic drugs. We employed survival assays, immunoblotting and microscopy for these studies.

**Results:**

We demonstrate that KCl, and to a lesser extent NaCl and RbCl can suppress the sensitivity of *S*. *pombe* to a wide range of antibiotics. Ammonium chloride and potassium hydrogen sulphate also suppressed drug sensitivity. This effect appears to depend in part on changes to membrane polarization and membrane transport proteins. Interestingly, we have found little relationship between the suppressive effect of KCl on sensitivity and the structure, polarity or solubility of the various compounds investigated.

**Conclusions:**

High concentrations of external potassium and other cations suppress sensitivity to a wide range of drugs in *S*. *pombe*. Potassium-rich environments may thus provide *S*. *pombe* a competitive advantage in nature. Modulating potassium ion homeostasis may sensitize pathogenic fungi to antifungal agents.

## Background

Understanding the complex relationship between K^+^ homeostasis and multidrug/cation sensitivity has important implications for a wide range of fields including microbial ecology, evolution, comparative genomics, fermentation and brewing, food spoilage, the treatment of infectious diseases and cancer therapy [1–8]. The fission yeast *Schizosaccharomyces pombe* utilizes an extensive repertoire of transporters and signaling pathways to regulate K^+^ homeostasis [9]. Proper maintenance of K^+^ homeostasis in turn plays an important role in regulating membrane potential and therefore resistance to cations, antibiotics such as hygromycin B, and chemotherapeutic agents in *S*. *pombe* [8]. In particular, expression of the Trk1 and Trk2 K^+^ transporters and the Hal4 kinase have been shown to be key regulators of K^+^ import in *S*. *pombe* and other yeasts [9, 10]. *hal4* or *trk1*Δ *trk2*Δ double mutants fail to adequately import K^+^, resulting in membrane hyperpolarization and multidrug sensitivity [8]. Addition of excess KCl to the media partially restored the resistance of these mutants to cations and multiple drugs. These genes have thus been proposed to facilitate resistance to multiple drugs in *S*. *pombe* [8]. Conversely, yeast mutants unable to effectively regulate H^+^ or K^+^ ion efflux have depolarized cell membranes and increased resistance to cations and hygromycin B [11–13]. Similarly, membrane depolarization by addition of excess KCl or NaCl has been shown to suppress the sensitivity of wild type *Saccharomyces cerevisiae* to hygromycin B. Together, these studies suggest that plasma membrane potential influences sensitivity to cations and cationic drugs in *S*. *pombe* and other yeasts. Yet, the precise relationship between drug resistance in *S*. *pombe* and external KCl concentrations (particularly in its natural habitats) remains unclear. Furthermore, the relative effects of external KCl and NaCl concentrations on drug sensitivity in *S*. *pombe* remain poorly characterized.

Despite its extensive characterization under laboratory conditions, little is known about how K^+^ homeostasis influences the survival of *S*. *pombe* in its natural environment. *S*. *pombe* has frequently been isolated from a restricted range of fermenting plant products rich in potassium [9, 14]. Under natural conditions, *S*. *pombe* must compete for resources with other micro-organisms including lactic acid bacteria (LAB), non-LAB bacteria, other fungi and yeasts [6, 7]. LAB produce lactic acid as a byproduct of fermentation. They also produce bacteriocins (peptide antimicrobials) and other bacteriostatic molecules [5–7, 15]. These substances are believed to confer competitive advantages to LAB in their natural environment [6]. Additionally *S*. *pombe* must also be able to resist the potential effects of yeast killer toxins [16, 17], acetic acid [7] and antibiotics produced by non-LAB strains (e.g. *Streptomyces spp*.) [7, 18]. Studies on the microbial dynamics of fermenting millet and wine, both rich in potassium, suggest that *S*. *pombe* is particularly well adapted to its natural environment(s) [6]. As such, it must be able to withstand not only high external potassium levels and low pH but also a diverse range of antimicrobial substances. Wild type (wt) *S*. *pombe* strains are however relatively sensitive to several antibiotic and chemotherapeutic substances under standard laboratory conditions [8]. In both yeast and bacteria, such sensitivity is closely linked to the proper regulation of the plasma membrane potential [4, 8, 13, 19]. Cation homeostasis and osmoregulation have also been linked to drug resistance in *S*. *cerevisiae*, *Candida albicans* (*C*. *albicans*) and *Aspergillus spp*. [11, 20–22]. The development of resistance to antifungal therapeutics continues to limit their clinical efficacy [21, 23, 24]. Understanding the link between ion homeostasis, osmoregulation and drug resistance may thus lead to the development of new treatment strategies. In a previous study [25] we noted that increasing the concentration of external K^+^ in media greatly suppressed the sensitivity of *S*. *pombe* to the antibiotics bleomycin and phleomycin. Since *S*. *pombe* can tolerate a wide range of external K^+^ concentrations which in turn affect plasma membrane polarization [8, 9], we hypothesized that potassium-rich natural environments may confer a competitive advantage to this and other yeast species.

In the present study, we have investigated the effect of external K^+^ concentrations on the sensitivity of *S*. *pombe* to a wide range of antibiotics, other antimicrobial agents and chemotherapeutic agents. In addition, we have also compared and contrasted the relative effects of KCl, its analogue RbCl, and NaCl on drug sensitivity in this yeast. We demonstrate that KCl and to a lesser extent NaCl and RbCl can suppress the sensitivity of *S*. *pombe* to a wide range of antibiotics. We also demonstrate that high external ammonium concentrations similarly suppress drug sensitivity in *S*. *pombe*. Interestingly, we have found little relationship between the suppressive effect of KCl on sensitivity and the structure, polarity or solubility of the various compounds investigated. Together, our findings suggest that low pH, high KCl, environments may provide a unique environmental niche for *S*. *pombe* in nature.

## Materials and Methods

### Drugs

All drugs except caspofungin were from Sigma Aldrich (Sigma Aldrich AB, Stockholm, Sweden). Caspofungin was from Santa Cruz Biotechnology (Heidelberg, Germany) All drugs were dissolved in either dH_2_O, dimethyl sulphoxide (DMSO) or ethanol and stored at -20°C.

### Growth of *S*. *pombe* strains

*S*. *pombe* was cultured in YES medium [26] at 30°C unless otherwise indicated. For potassium free media (HGA media), we used a solution of 3% glucose and 0.05% NH_4_Cl in dH_2_O. pH was adjusted to 6.3 with 5% ammonia solution. Strains are listed in Table 1.

**Table 1:** *S*. *pombe* strains used in this study.

*h^-^ L972*	Lab stock
*h^-^ rad3::ura4^+^ ura4-D18 leu1–32* (FY7799)	YGRC
*h^-^ rad24::ura4^+^ leu1 ura4-D18 ade6-M210* (FY13517)	YGRC
*h^-^ sty1::ura4^+^ ura4-D18*	Lab stock
*h^-^ chk1::3XHA ade6–216 leu1–32*	N. Walworth
*h^-^ hal4::ura^+^ ura4- D18*	K. Shiozaki
*h^+^ trk1::kanR trk2::kanR*	K. Shiozaki
*h^-^ pzh1::ura4^+^ ade6-M210 ura4-D18 leu1–32*	J. Ariño
*h^-^ sod2::ura4^+^ ade6-M210 ura4-D18 leu1–32*	J. Ariño

YGRC, Yeast Genetic Resource Center, Osaka, Japan

### Drug sensitivity assays

Log phase cultures were resuspended in fresh media containing the desired drug with or without potassium chloride. After the required incubation period, the cultures were equilibrated for cell number, serially diluted and spotted unto YES agar plates. The plates were incubated at 30°C for 2–3 days. Alternatively, log phase cultures were serially diluted and spotted unto YES agar plates containing the desired alkali salt or drug concentrations and incubated at 30°C for 2–3 days.

### Immunoblotting

Monoclonal antibodies directed against HA were from Santa Cruz Biotechnology (Heidelberg, Germany). Mouse monoclonal antibodies directed against phospho-(Thr180/Tyr182) p38 were from Cell Signaling Technology (Bionordika (Sweden) AB, Stockholm, Sweden). Monoclonal antibodies directed against α-tubulin were from Sigma-Aldrich (Sigma Aldrich AB). For immunoblotting, protein extracts were prepared as previously described [25] with addition of 1 × PhosStop phosphatase inhibitor cocktail (Roche Diagnostics Scandinavia AB, Bromma, Sweden). Proteins were separated by SDS-PAGE. Epitope-tagged proteins were detected with the appropriate monoclonal antibodies.

### Microscopy

Cells were harvested, fixed in 70% ethanol and stored at 4°C until analyzed. Images were obtained with a Zeiss AxioCam on a Zeiss Axioplan 2 microscope with a 100 × objective using a 4,6-diamidino-2-phenylindole (DAPI) filter set. Fixed cells were mounted in VECTASHIELD mounting medium and visualized using differential interference contrast (DIC) or a DAPI filter set. For studies with doxorubicin, cells were pelleted and directly examined by fluorescence microscopy.

## Results

### Potassium chloride suppresses drug sensitivity in *S*. *pombe*

**Fig 1:**
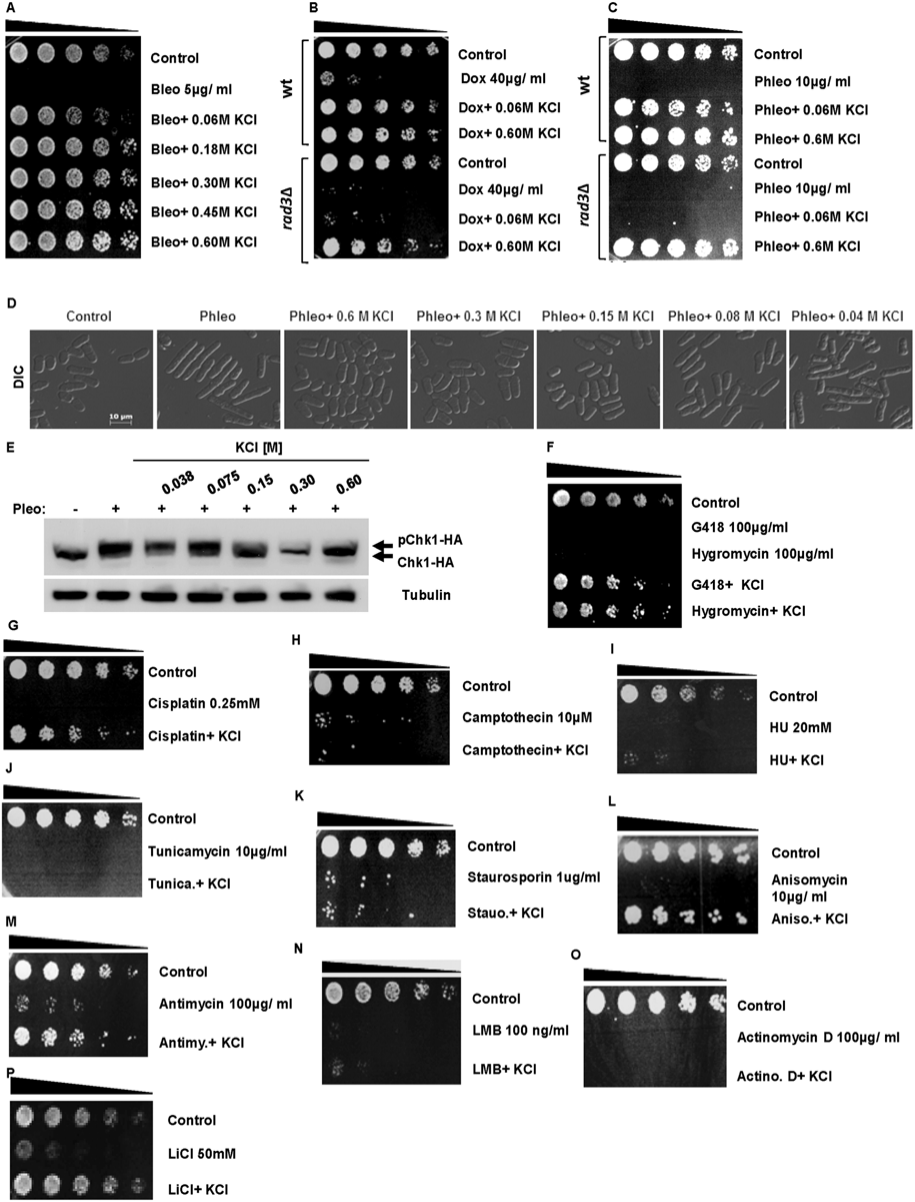
KCl suppresses drug sensitivity in *S*. *pombe* **A.** Wild type (wt) *S*. *pombe* cells were cultured in the presence of the 5 μg/ ml bleomycin alone or with indicated concentrations of KCl in the media for 24 h at 30°C. Equal cell numbers were serially diluted and plated on YES agar. Plates were incubated at 30°C for 2–3 days. **B.** Wt and *rad3*Δ mutants were exposed to 40 μg/ ml doxorubicin alone or with the indicated concentrations of KCl in the media for 24 h at 30°C and treated as in A. **C.** Wt and *rad3*Δ cells were treated as in B, except that the cells were exposed to 10 μg/ ml phleomycin. **D.** Wt *S*. *pombe* cells were incubated with 10 μg/ ml phleomycin alone or with the indicated concentrations of KCl in the media. Cells were fixed in 70% ethanol and examined by microscopy. **E.** A strain expressing HA- tagged Chk1 was incubated with 10 μg/ ml phleomycin in the presence of the indicated KCl concentrations. Total lysates were resolved by SDS- PAGE and probed with antibodies directed against HA. Tubulin was used to monitor equal gel loading. **F.** Wt cells were exposed to 100 μg/ ml of G418 or hygromycin for 24 h with or without 0.6 M KCl and then treated as in A. **G- P.** Wt cells were exposed to the indicated drugs with or without 0.6 M KCl for 24 h and treated as in A.

In order determine the effect of external K^+^ concentrations on drug sensitivity, *S*. *pombe* cells were exposed to the antibiotic DNA damaging agents bleomycin (5 μg/ ml), doxorubicin (400 nM) or phleomycin (10 μg/ ml) in the presence of increasing KCl concentrations. KCl effectively suppressed the sensitivity of wt *S*. *pombe* cells to bleomycin at concentrations as low as 60 mM (Fig. 1A). At these concentrations, KCl also completely suppressed the sensitivity of wt *S*. *pombe* cells to doxorubicin and phleomycin (Fig. 1B and 1C). We noted, however, that 60 mM KCl was insufficient to suppress the sensitivity of the checkpoint-deficient *rad3*Δ and *rad24*Δ mutants to doxorubicin and phleomycin (Fig. 1B, C, and S1A, F Fig.). Microscopic examination of wt cells exposed to phleomycin suggested that low doses of KCl do not prevent DNA damage induced by this agent (Fig. 1D). Activation of the Rad3- regulated DNA response pathway results in *S*. *pombe* induces Chk1 phosphorylation observable as a band shift on Western blots [27]. In agreement, the complete abolition of phleomycin-induced Chk1 phosphorylation was observed only at concentrations of 0.3 to 0.6 M KCl (Fig. 1E). Exposure to 0.6 M KCl in the presence of the aminoglycoside antibiotics G418 and hygromycin similarly abolished sensitivity of wt *S*. *pombe* to these agents (Fig. 1F). At 0.6 M, KCl also suppressed the sensitivity of wt *S*. *pombe* cells to cisplatin, anisomycin, and antimycin A (Fig. 1G, L, and M). In contrast, KCl did not abolish sensitivity to camptothecin, hydroxyurea (HU), tunicamycin, staurosporin, leptomycin B (LMB) or actinomycin D (Table 2, Fig. 1H-K, N, and O). Furthermore, KCl did not inhibit the effects of latrunculin B and methylbenzimidazol-2yl carbamate (MBC) on cytokinesis and mitosis respectively (S1G Fig.). Co- exposure to 0.6 M KCl also suppressed sensitivity to 50 mM LiCl (Fig. 1P). The suppressive effect of KCl on phleomycin sensitivity to was not due to inactivation of the drug. In fact, pre-incubation in KCl of the drug alone greatly enhanced the cytotoxic effect of phleomycin in wt and *rad24*Δ mutants (S1E Fig.).

**Table 2:** List of drugs investigated in this study.

Drug	Class	Cellular activity	Solubility	KCl—induced resistance^a^
Bleomycin	Glycopeptide antibiotic	DNA double strand breaks	H_2_0	YES
Phleomycin	Glycopeptide antibiotic	DNA double strand breaks	H_2_0	YES
G418	Aminoglycoside antibiotic	Inhibition of protein synthesis	H_2_0	YES
Hygromycin B	Aminoglycoside antibiotic	Inhibition of protein synthesis	H_2_0	YES
Doxorubicin	Anthracycline antibiotic	DNA strand intercalation/	H_2_0	YES
Anisomycin	Antibiotic	Inhibition of protein synthesis	DMSO	YES
Staurosporine	Alkaloid antibiotic	Protein kinase inhibiton	DMSO	NO
Tunicamycin	Nucleoside antibiotic	Inhibition of protein N- glycosylation	DMSO	NO
Leptomycin B	Secondary metabolite antibiotic	Crm1/ nuclear export inhibitor	Ethanol	NO
Antimycin A	Secondary metabolite antibiotic	Inhibition of ATP synthesis	Ethanol	YES
Actinomycin D	Polypeptide antibiotic	Inhibitor of transcription	DMSO	NO
Nigericin	Polyether antibiotic	Potassium, hydrogen and lead selective ionophore	Ethanol	YES
Valinomycin	Dodecadepsipeptide antibiotic	Potassium selective ionophore	DMSO	NO^b^
Caspofungin	Lipopeptide echinocandin	Cell wall disruption	H_2_0	YES
Amphotericin B	Polyene antimycotic	Cell wall disruption	DMSO	YES/ NO^c^
Nystatin	Polyene antimycotic	Cell wall disruption	DMSO	YES/ NO^d^
Clotrimazole	Antifungal azole	Cell wall disruption	DMSO	NO
Cisplatin	Platinum based anti- cancer agent	Induction of DNA cross- links	DMSO	YES
Camptothecin	Quinoline alkaloid	Topoisomerase 1 inhibiton	DMSO	NO
Carbendazim (MBC)	Benzimidazole	Inhibition of tubulin polymerization	DMSO	NO
Latrunculin B	Toxin from isolated from sponges	Inhibition of actin polymerization	DMSO	NO
Hydroxyurea	Anti- cancer agent	Deoxynucleotide synthesis inhibitor	H_2_0	NO

^a^ Refers to the ability of high external KCl concentrations to suppress drug sensitivity.

^b^ 0.6 M KCl enhanced sensitivity to sub-lethal concentrations of valinomycin.

^c^ Sensitivity to amphotericin B was suppressed by 0.06 M KCl and increased by 0.6 M KCl.

^d^ 0.6 M KCl enhanced sensitivity to sub-lethal concentrations of nystatin.

### Cations suppress drug sensitivity in *S*. *pombe*

**Fig 2:**
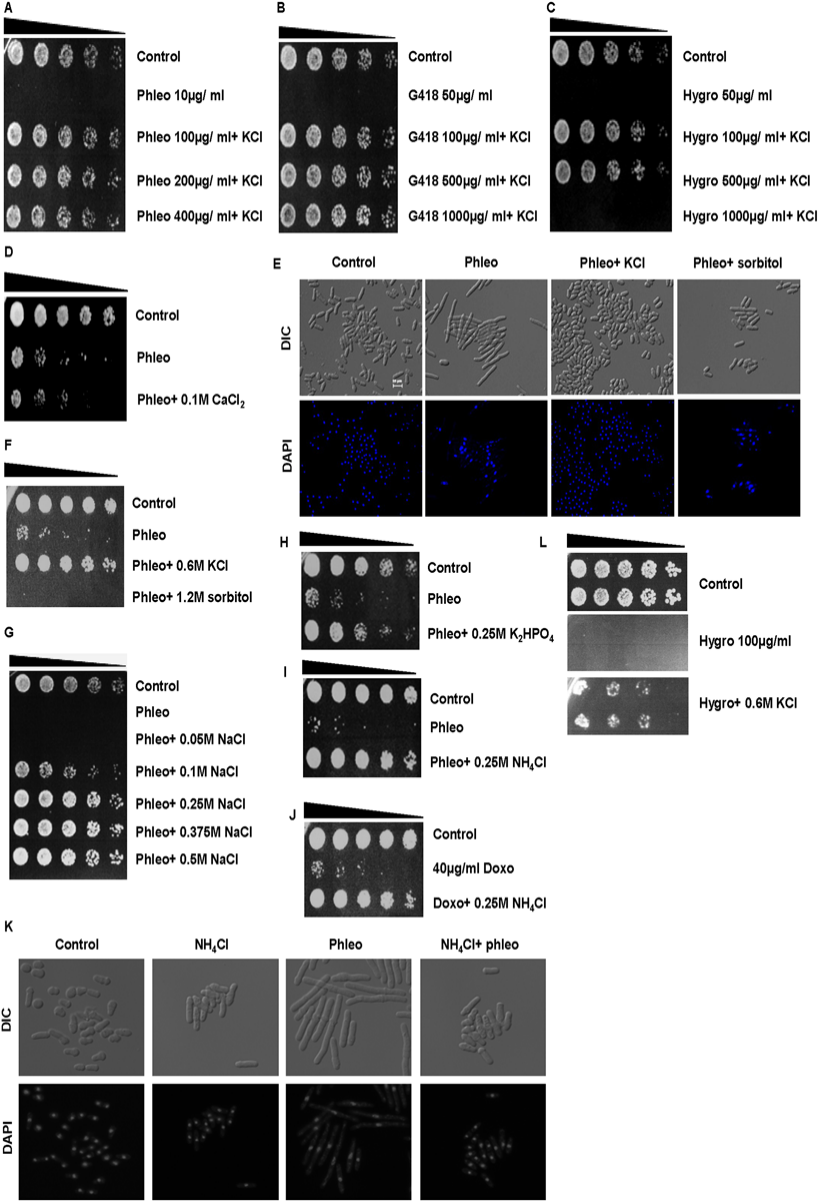
Suppression of drug sensitivity in *S*. *pombe* by alkali metal ions. **A- C.** Wt *S*. *pombe* cells were incubated with various concentrations of phleomycin (phleo), G418 and hygromycin (hygro) ± 0.6 M KCl for 24 h at 30°C. Equal cell numbers were serially diluted and plated on YES agar. Plates were incubated at 30°C for 2–3 days. **D.** Wt *S*. *pombe* cells were incubated with 10 μg/ ml phleomycin ± 0.1 M CaCl_2_ for 4 h at 30°C and treated as in A. **E.** Wt S. pombe cells were exposed to 10 μg/ ml phleomycin ± 0.6 M KCl or 1.2 M sorbitol for 6 h, fixed in 70% ethanol, stained with DAPI and examined by microscopy. **F.** Wt S. pombe cells were treated as in E. Equal cell numbers were serially diluted and plated on YES agar. Plates were incubated at 30°C for 2–3 days. **G.** Wt *S*. *pombe* cells were incubated with 10 μg/ ml phleomycin or in the presence of the indicated NaCl concentrations for 24 h at 30°C and treated as in A. **H.** Wt cells were incubated with 10 μg/ ml phleomycin ± 0.25 M K_2_HPO for 4 h at 30°C and treated as in A. **I.** Wt cells were incubated with 10 μg/ ml phleomycin ± 0.25 M NH_4_Cl for 24 h at 30°C and treated as in A. **H. I.** *S*. *pombe* cells were incubated with 0.6 M RbCl ± 0.6 M KCl for 24 h at 30°C. **J.** *S*. *pombe* cells were incubated with 40 μg/ ml doxorubicin ± 1 M NH_4_Cl for 24 h at 30°C. **K.** Cells were treated as in I, fixed in 70% ethanol, stained with DAPI and examined by microscopy. **L.** Log phase wt cultures were serially diluted and plated on YES agar containing 100 μg/ ml hygromycin ± 0.6 M KCl for 72 h at 30°C.

To further understand the effect(s) of KCl on drug sensitivity, we investigated the degree to which KCl suppresses sensitivity to phleomycin, G418 and hygromycin. In the presence of 0.6 M KCl, however, doses as high as 400 μg/ ml phleomycin, 1000 μg/ ml G418, and 500 μg/ ml hygromycin did not affect the survival of wild type *S*. *pombe* cells (Fig. 2A-C). At a concentration of 0.6 M therefore, KCl increased resistance to phleomycin, G418 and hygromycin by a factor of 400, 200, and 50 times respectively (Table 3). The minimum inhibitory concentrations (MICs) in our assays were 1 μg/ ml, 5 μg/ ml and 10 μg/ ml for phleomycin, G418 and hygromycin respectively (S1B,C Fig.). Calcium chloride (0.1 M) also failed to suppress drug sensitivity in *S*. *pombe* (Fig. 2D). Previous studies have similarly reported that sensitivity to bleomycin (structurally similar to phleomycin) in particular, is strongly influenced by internal K^+^ levels [8]. The ability of KCl to suppress sensitivity to these drugs did not result from the induction of osmotic stress, since equiosmotic concentrations of sorbitol did not have this effect (Fig. 2E, F and S2H Fig.). In contrast, sodium chloride (NaCl), ammonium chloride (NH_4_Cl), rubidium chloride (RbCl) and potassium hydrogen phosphate (K_2_HPO_4_) all suppressed drug sensitivity (Fig. 2G-I, K, S2A, B Fig.). The ability of NH_4_Cl to suppress drug sensitivity was not limited to phleomycin since it also suppressed sensitivity to doxorubicin (Fig. 2J). The observation that K_2_HPO_4_ suppressed drug sensitivity in *S*. *pombe* ruled out a role for chloride (Cl^-^) ions in this activity (Fig. 2H). Similarly, the ability of NH_4_Cl to suppress drug sensitivity suggested that this activity is not restricted to metal ions (Fig. 2I-K, S2B Fig.). Under these conditions, KCl also facilitated the survival of *S*. *pombe* in the presence of 100 μg/ ml G418 and hygromycin (Fig. 2L). The ability of KCl, NaCl and RbCl to suppress drug sensitivity was not strictly identical. We noted that 0.6 M KCl suppressed the sensitivity of *S*. *pombe* to 1 mM but not 2 mM cadmium chloride (CdCl_2_) (S2C Fig.). In contrast, co-culture in the presence of 0.5 M NaCl suppressed the sensitivity to 2 mM CdCl_2_ (S2D Fig.). *S*. *pombe* cells grew poorly in liquid YES media in the presence of 0.6 M NaCl. KCl (0.6 M) did not suppress sensitivity to 0.4% acetic acid (S2F Fig.). The ability of KCl to suppress hygromycin and phleomycin sensitivity was also not affected by the presence of 0.1%- 0.3% acetic or lactic acid (S2G Fig.). Furthermore, acidification of the external pH with lactic acid (pH 6.4–3.1) had little or no effect on viability (S2G Fig.).

**Table 3:** Relative effect of external KCl on drug sensitivity in *S*. *pombe*.

Drug	MIC (μg/ ml)	MIC 0.6M KCl (μg/ ml)	Fold increase in resistance
Phleomycin	1.0	>400	>400
G418	10.0	>1000	>100
Hygromycin B	10.0	500	50

### KCl suppresses drug sensitivity independently of Sty1, Hal4, and Trk1/2

**Fig 3:**
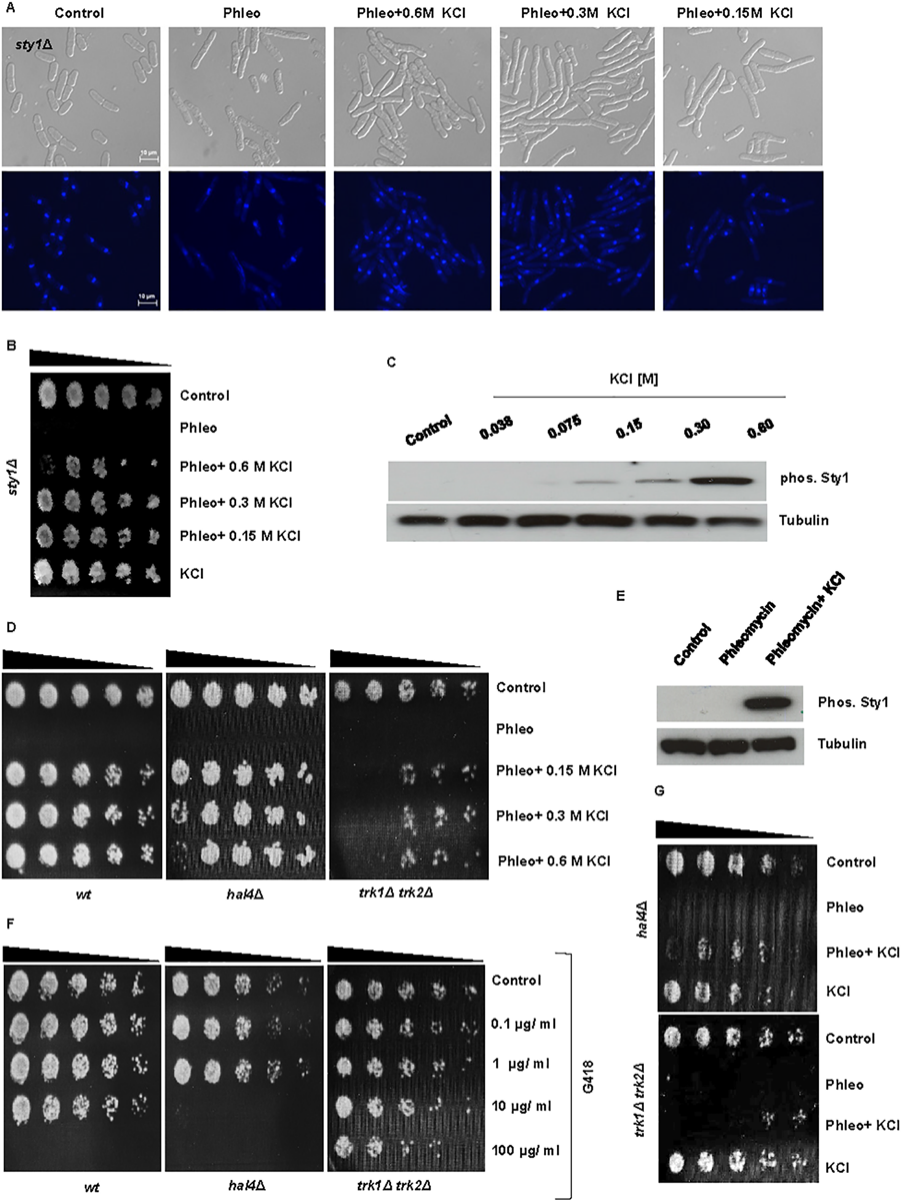
Sty1 is not required for the suppressive effect of KCl on drug sensitivity. **A.** sty1Δ mutants were incubated with 10 μg/ ml phleomycin ± the indicated concentration of KCl for 4 h. Cells were fixed in 70% ethanol, stained with DAPI and examined by microscopy. **B.** Cell were treated as in A for 4 h, serially diluted on YES plates and incubated for 2–3 days at 30°C. As an extra control, the mutant was also exposed to 0.6 M KCl alone. **C.** Wt *S*. *pombe* cells were incubated with the indicated concentrations of KCl for 10 min at 30°C. Total lysates were resolved by SDS- PAGE and probed with antibodies directed against phos. p38. Tubulin was used to monitor equal gel loading. **D.** Wt, *hal4*Δ and *trk1*Δ *trk2*Δ cells were exposed to 10 μg/ ml phleomycin ± the indicated concentrations of KCl for 4 h at 30°C and treated as in B. **E.** Wt *S*. *pombe* cells were incubated with 10 μg/ ml phleomycin ± 0.6 M KCl. Total lysates were treated as in C. **F.** Wt, *hal4*Δ and *trk1*Δ *trk2*Δ cells were exposed to increasing doses of G418 for 4 h and treated as in B. **G.** *hal4*Δ and *trk1*Δ *trk2*Δ cells were exposed to 10 μg/ ml phleomycin ± 0.6 M KCl or o.6 M KCl alone and treated as in B.

The MAPK Sty1 plays a central role in mediating resistance to environmental stresses in *S*. *pombe* [28]. Sty1 has also been shown to regulate the Hal4 kinase, which together with the Trk1 and Trk2 transporters regulates K^+^ uptake in *S*. *pombe* [8, 29]. Sty1 was not required for KCl- mediated suppression of phleomycin sensitivity in *S*. *pombe* (Fig. 3A and 3B). In contrast to wt cells however, *sty1*Δ mutants displayed an elongated phenotype when exposed to phleomycin in the presence of 0.15–0.6 M KCl (Figs. 2E and 3A). Furthermore, lower concentrations of KCl (0.15b –0.3 M) were more effective at suppressing phleomycin sensitivity than higher concentrations (0.6 M) in this mutant (Fig. 3B). Immunoblotting demonstrated only minimal activation of Sty1 at concentrations of KCl (0.04–0.3 M) sufficient to suppress sensitivity to phleomycin (Fig. 3C). In addition, *sty1*Δ mutants grew worse in the presence of phleomycin and 0.15–0.6 M KCl than in the presence of 0.6 M KCl alone (Fig. 3B). Exposure to phleomycin alone did not induce Sty1 activation (Fig. 3E). In our study, the sensitivity of *sty1*Δ mutants to G418 was not greater than observed for wt cells (S3A Fig.). Together, our observations suggest that Sty1 is not required for KCl- mediated suppression of phleomycin *per se*. Sty1 does seem to enhance the survival of *S*. *pombe* cells however, when exposed to the combined stresses of phleomycin and KCl exposure (Fig. 3B) [28].

Trk1 and Trk2, together with Hal4, regulate K^+^ uptake in *S*. *pombe* [8, 30]. Mutants lacking *trk1* ^*+*^ and *trk2* ^*+*^, or *hal4* ^*+*^, display membrane hyperpolarization and sensitivity to cations and cationic drugs [8]. As previously reported [8], high external K^+^ concentrations suppressed the sensitivity of *hal4*Δ mutants to cationic drugs. In our study, 0.3 M KCl suppressed sensitivity to 10 μg/ml phleomycin to the same degree in wt and *hal4*Δ mutants (Fig. 3D). Interestingly, higher concentrations (0.6 M) of KCl appeared to be less effective at suppressing the sensitivity of *hal4*Δ mutants to phleomycin (Fig. 3D). Co-exposure to KCl also suppressed the sensitivity of *trk1*Δ *trk2*Δ double mutants to phleomycin, albeit less efficiently than in wt and *hal4*Δ mutants (Fig. 3D). When cultured in the presence of 0.6 M KCl, we noted little or no effect on the viability of *hal4*Δ and *trk1*Δ *trk2*Δ mutants (Fig. 3G). The reduced viability observed following exposure to KCl and phleomycin may be due to the inability of these mutants to import sufficient levels of K^+^. We also observed that while *hal4*Δ mutants were more sensitive to G418 than wt cells, *trk1*Δ *trk2*Δ mutants were more resistant than *hal4*Δ mutants to this agent (Fig. 3F). We also observed that Hal4 was not required for KCl-mediated suppression of LiCl sensitivity (S3E Fig.). Factors other than membrane polarity are thus likely to influence sensitivity to particular drugs in *S*. *pombe* in the presence of medium to high KCl concentrations. The Na^+^ (Li^+^) / H^+^ antiporter Sod2 was not required for KCl-mediated suppression of LiCl and phleomycin sensitivity in *S*. *pombe* (S3B,E Fig.).

### KCl inhibits drug import in *S*. *pombe*

**Fig 4:**
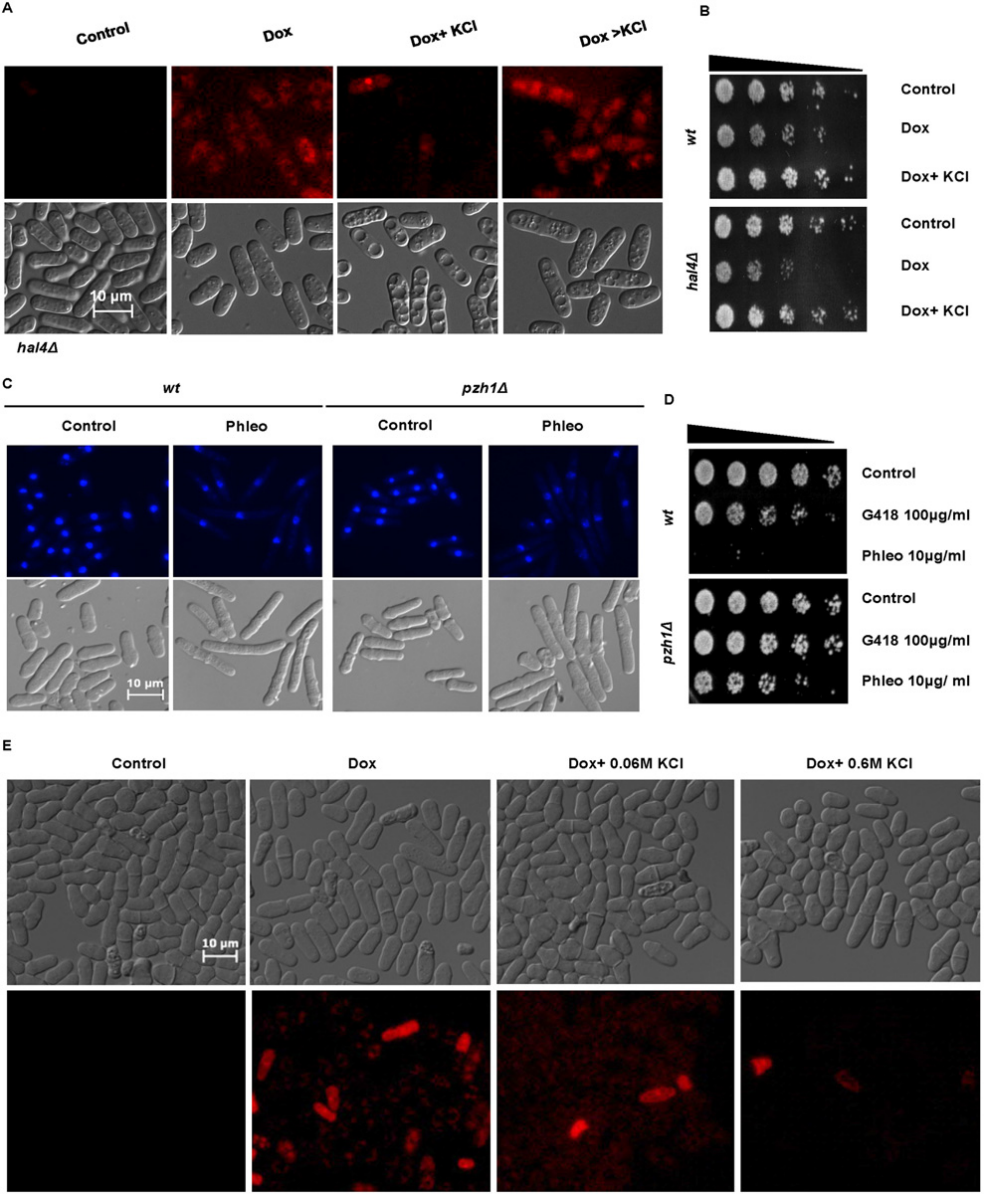
KCl blocks doxorubicin uptake in *S*. *pombe*. **A.** *hal4*Δ mutants were exposed to 40 μg/ ml doxorubicin alone and together with 0.6 M KCl for 4 h or with doxorubicin for 2 h followed by coexposure to doxorubicin and KCl for another 2 h and examined by microscopy. **B.** Wt and *hal4*Δ mutants were treated as in A for 4 h, serially diluted and plated unto YES agar. Plates were incubated at 30°C for 2–3 days. **C.** Wt and *pzh1*Δ mutants were exposed to 10 μg/ ml phleomycin ± 0.6 M KCl for 4h, fixed in 70% ethanol and examined by microscopy. **D.** Wt and *pzh1*Δ mutants were exposed to 10 μg/ ml G418 or 5 μg/ ml phleomycin for 4 h and treated as in B. **E.** Wt cells were treated with 40 μg/ ml doxorubicin alone or together with 0.06M or 0.6 M KCl for 2 h.

Mutants lacking *hal4* ^*+*^ have previously been shown to import higher levels of doxorubicin than wt cells [8]. In our study, co-exposure to KCl (0.6 M) and doxorubicin blocked the import of the drug. In marked contrast however, culture in the presence of KCl following exposure to doxorubicin did not result in the efflux of the drug (Fig. 4A). Previous studies have demonstrated the increased uptake of cationic compounds by *S*. *pombe* cells with hyperpolarized membranes [8]. Microscopic analyses clearly demonstrated that co-exposure to KCl inhibited the uptake of doxorubicin compared to cells exposed to the latter alone (Fig. 4A panels 2 and 3). Additional analyses demonstrated that the ability of KCl to prevent the import of doxorubicin into the cell was concentration-dependent (Fig. 4E). Furthermore, the ability of KCl to inhibit doxorubicin import correlated with its ability to suppress sensitivity to the drug in *hal4*Δ mutants (Fig. 4B). In contrast, exposure to 1.2 M sorbitol, did not prevent doxorubicin uptake (S2H Fig.). Mutants lacking the phosphatase Pzh1 are unable to effectively export K^+^ ions from interior of the cell. High internal K^+^ levels result in sensitivity to this ion, but resistance to Na^+^ [12]. Following exposure to phleomycin, *pzh1*Δ mutants appeared similar to wt cells (Fig. 4C). Survival assays demonstrated however, that *pzh1*Δ mutants were slightly more resistant to G418 than wt cells (Fig. 4D). Furthermore, *pzh1*Δ mutants were significantly more resistant to phleomycin than wt cells (Fig. 4D). Following long term exposure to phleomycin however, co-exposure to KCl suppressed sensitivity in a manner similar to that observed in wt cells (S3C,D Fig.). The relative KCl concentration-dependent sensitivity of *pzh1*Δ mutants to phleomycin indicated that their internal K^+^ ion levels are insufficient to block the activity of the drug. Our finding that *pzh1*Δ mutants were relatively more resistant to phleomycin than G418 provided further evidence for the exquisite sensitivity of bleomycin and phleomycin to internal K^+^ concentrations.

**Fig 5:**
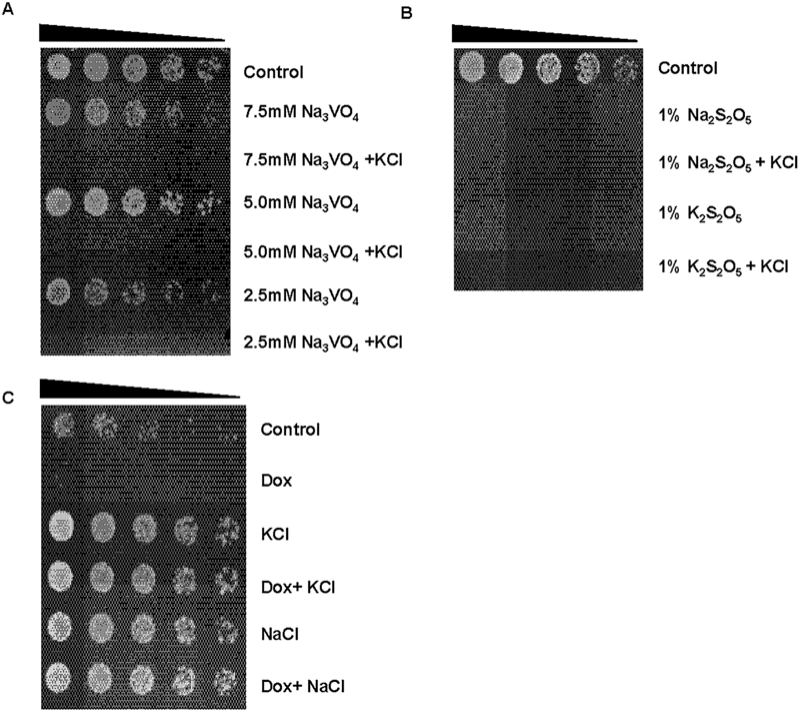
KCl enhances sensitivity to Na_3_VO_4_ in *S*. *pombe*. **A.** Wt cells were incubated with 7.5 mM of Na_3_VO_4_ ± 0.6 M KCl for 24 h, serially diluted and plated unto YES agar. Plates were incubated at 30°C for 2–3 days. **B.** Wt cells were incubated with 1% Na_2_S_2_O_5_ or 1% Na_2_S_2_O_5_ ± 0.6 M KCl and treated as in A. **C.** Wt cells were incubated in HGA- medium (see Materials and methods) with phleomycin ± KCl or NaCl and treated as in A.

The findings above provided additional evidence for KCl-induced membrane depolarization in modulating the sensitivity of *S*. *pombe* to cationic drugs [8]. To further test this hypothesis, we investigated the effect of KCl on sensitivity to sodium orthovanadate (Na_3_VO_4_). The negatively charged VO_4_^3-^ acts as an inhibitor of protein tyrosine and alkaline phosphatases. As predicted, KCl significantly enhanced the toxicity of Na_3_VO_4_ (Fig. 5A). In addition, KCl had no effect on sensitivity to 1% potassium metabisulfite (K_2_S_2_O_5_) or 1% sodium metabisulfite (Na_2_S_2_O_5_) in *S*. *pombe* (Fig. 5B). To further investigate the role for potassium and sodium in suppressing drug sensitivity, we investigated their activity in HGA medium (3% glucose and 0.05% NH_4_Cl in dH_2_O. pH was adjusted to 6.3 with 5% ammonia solution). Taken together, these experiments demonstrated that high external concentrations of KCl and NaCl are sufficient to suppress drug sensitivity in *S*. *pombe* (Fig. 5C).

### Effect of KCl on fungicide and antiporter sensitivity

**Fig 6:**
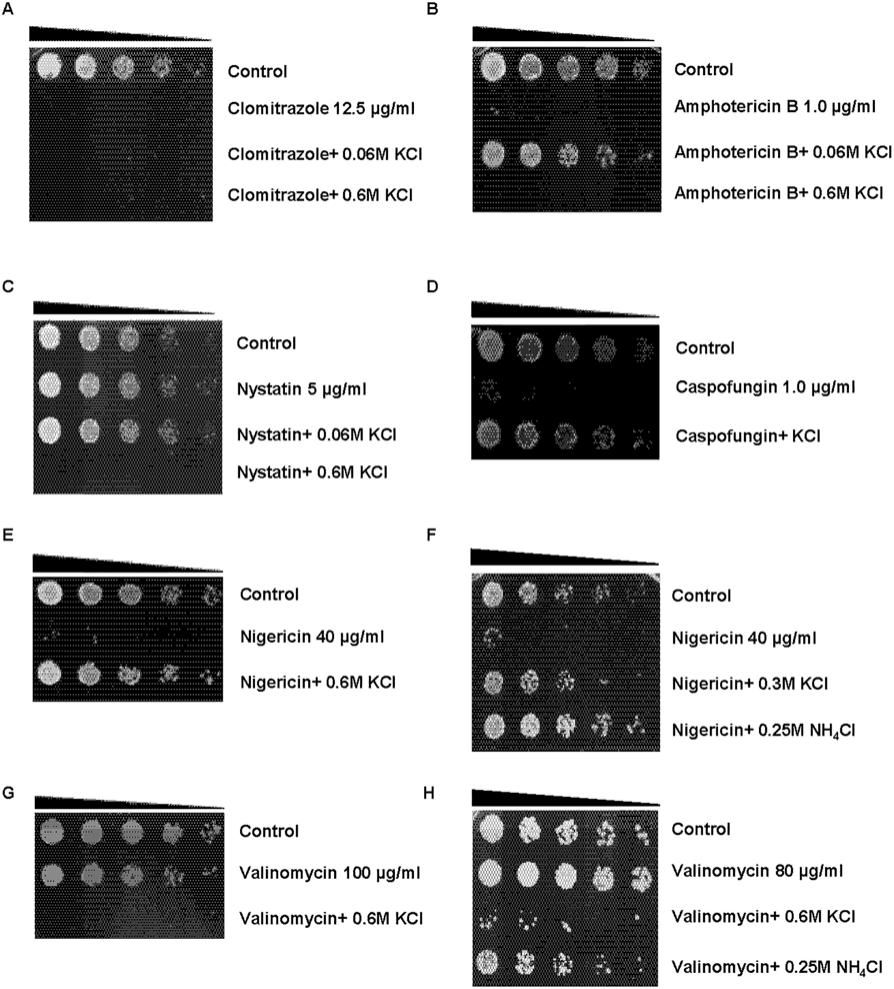
Effect of KCl on *S*. *pombe* sensitivity to fungicides. **A- C.** Wt cells were exposed to 10 μg/ ml clomitrazole, 1 μg/ ml amphotericin B or 5 μg/ ml nystatin alone and together with the indicated concentrations of KCl for 24 h, serially diluted and plated unto YES agar. Plates were incubated at 30°C for 2–3 days. **D- E.** Wt cells were exposed to 1 μg/ ml caspofungin or 40 μg/ ml nigericin ± 0.6 M KCl and treated as in A. **F.** Wt cells were exposed to 20 μg/ ml nigericin ± 0.3 M KCl or 0.25 M NH_4_Cl for 24 h and treated as in A. **G.** Wt cells were exposed to 100 μg/ ml valinomycin ± 0.6 M KCl for 24 h and treated as in A. **H.** Wt cells were exposed to 80 μg/ ml valinomycin ± 0.6 M KCl or 0.25 M NH_4_Cl for 24 h and treated as in A.

We next investigated the effect of high external KCl concentrations on drugs that disrupt fungal cell membranes or cation homeostasis (ionophores). Clotrimazole disrupt fungal cell membranes by inhibiting ergosterol synthesis, resulting in the leakage of ions and small molecules from the cell [31, 32]. KCl did not protect *S*. *pombe* from the lethal effects of 15 μg/ ml clotrimazole at either 0.06 or 0.6 M (Fig. 6A). Clotrimazole is an organic compound and this might account for the inability of KCl to inhibit its activity. In contrast to clotrimazole, polyene antifungal drugs such as amphotericin B and nystatin bind directly to ergosterol, leading the formation of pores in the membrane and the leakage of ions and small molecules from the cell [33, 34]. In addition, amphotericin B may also inhibit the Na^+^/ K^+^ pump contributing to cell death [35]. Interestingly, KCl exerted differential effects on the sensitivity of *S*. *pombe* to these drugs. Co-exposure to 0.06 M KCl inhibited the lethal effects of 1μg/ ml amphotericin B (Fig. 6B). In stark contrast, this suppression of sensitivity was not observed when wt *S*. *pombe* cells were co-exposed to amphotericin B and 0.6 M KCl (Fig. 6B). Co exposure to 3 μg/ ml nystatin and 0.06 M KCl did not affect sensitivity to this drug, while 0.6 M KCl enhanced sensitivity under similar conditions (Fig. 6C). Further analyses indicated that KCl similarly enhances sensitivity to amphotericin B at concentrations between 0.3 M and 0.6 M (S4A Fig.). Together, these findings demonstrated that KCl at concentrations of 0.15 M and above enhances sensitivity to the polyene antifungals amphotericin B and nystatin. It remains unclear if this KCl- induced increase in sensitivity is due to the non-ionic nature of these drugs. Caspofungin is an echinocandin antifungal drug that inhibits cell wall synthesis by inhibiting the enzyme (1→3)-β-D-glucan synthase [36]. In our studies, co-exposure to 0.6 M KCl completely inhibited sensitivity to 1 μg/ ml caspofungin (Fig. 6D). In contrast to its effect on the sensitivity of *S*. *pombe* to phleomycin (Fig. 2D, E), sorbitol (1.2 M) similarly abolished the lethal effects of caspofungin (S4B Fig.). Caspofungin is water soluble, suggesting that KCl may counter its activity by influencing membrane polarity. We next investigated the effect of KCl on sensitivity to the ionophore antibiotics nigericin and valinomycin [37, 38]. Nigericin is completely insoluble in water and functions as an antiporter for K^+^ and other ions, inducing cell death in part by causing acidification of the cytoplasm and ion leakage from the cell [38]. KCl (0.6 M) abolished sensitivity to 10 μg/ ml nigericin (Fig. 6E). Co-exposure to NH_4_Cl similarly suppressed sensitivity to nigericin. In contrast, 0.6 M KCl enhanced sensitivity to 100 μg/ ml valinomycin (Fig. 6F). Interestingly, exposure to 100 μg/ ml valinomycin alone, did not affect viability in *S*. *pombe*. Furthermore, co-exposure to NH_4_Cl did not induce sensitivity to this drug. Like nigericin, valinomycin is insoluble in water and induces K^+^ leakage from the cell. It thus remains unclear why KCl differentially affects the sensitivity of *S*. *pombe* to nigericin and valinomycin.

## Discussion

In this study, we investigated the effect of high external K^+^ and other ion concentrations on drug sensitivity in *S*. *pombe*. In *S*. *pombe* and other yeasts, a close relationship exists between the regulation of K^+^ etc. homeostasis and the polarity of the cell membrane. Deletion of *hal4* ^*+*^ or co-deletion of *trk1* ^*+*^ and *trk2* ^*+*^ results in membrane depolarization and sensitivity to cations [8]. Interestingly, mutants lacking *hal4* ^*+*^ or both *trk1* ^*+*^ and *trk2* ^*+*^ display hypersensitivity to a range of antibiotics, chemotherapeutic agents and other drugs [8]. These compounds, despite differences in structure and chemical classes, are all cationic. It has been proposed that the membrane hyperpolarization induced by deleting *hal4* ^*+*^ or *trk1* ^*+*^ and *trk2* ^*+*^ facilitates the import of these compounds into the cell. Elevating the external KCl concentration partially restored resistance to cations and drugs in *hal4*Δ and *trk1*Δ *trk2*Δ mutants [8]. Furthermore, external KCl and NaCl concentrations have been shown to suppress the sensitivity of *S*. *cerevisiae* to hygromycin [13]. The ability of elevated external KCl concentrations to suppress drug sensitivity in *hal4*Δ and *trk1*Δ *trk2*Δ mutants suggested to us that a similar effect might occur in wt *S*. *pombe*. We thus investigated the effect of elevating the external concentration of K^+^ and other ions on the sensitivity of *S*. *pombe* to various cations and drugs (Table 3 and S5 Fig.).

In general, elevating the external K^+^ concentration of the media to 0.3–0.6 M was sufficient to suppress the sensitivity of wt *S*. *pombe* to cationic drugs such as bleomycin, phleomycin, G418 and hygromycin. KCl was more effective at suppressing sensitivity to water soluble compounds. Nevertheless, KCl did not suppress sensitivity to hydroxyurea which is highly soluble in water. Conversely, co-exposure to KCl also suppressed sensitivity to anisomycin and antimycin which are nonpolar compounds. Thus, no clear relationship between that ability of KCl to suppress sensitivity and the solubility, polarity or class of drugs investigated in this study was identified (Fig. 1 and S5 Fig.). The ability of KCl to suppress drug sensitivity was dependent on the K^+^ ion and not hyperosmosis *per se*, as sorbitol did not suppress drug sensitivity in a similar manner. The ability of metal cations to suppress drug sensitivity in *S*. *pombe* was not restricted to K^+^ since alkali cations Na^+^ and Rb^+^ also suppressed drug sensitivity in *S*. *pombe*. Indeed, co-exposure to NH_4_Cl also suppressed drug sensitivity. The observation that K_2_HPO_4_ similarly suppressed drug sensitivity ruled out a role for Cl^-^ ions. Furthermore, the activity of these cations on cell survival was not strictly identical since Na^+^ was more effective at suppressing sensitivity to cadmium than K^+^. Interestingly, K^+^ facilitated the growth of *S*. *pombe* on solid rich media in the presence of NaCl. In terms of relative survival and culture mass, K^+^ was the most effective suppressor of drug sensitivity. The degree to which KCl suppressed drug sensitivity in *S*. *pombe* also varied amongst bleomycin, phleomycin, G418 and hygromycin. A previous study suggested that the deletion of *hal4* ^*+*^ or *trk1* ^*+*^ and *trk2* ^*+*^ enhanced sensitivity to bleomycin, to a greater degree than other drugs tested [8]. In our study, KCl was similarly most effective at inhibiting the sensitivity of wt *S*. *pombe* cells to bleomycin and phleomycin. KCl also suppressed sensitivity to hygromycin to a greater degree than to G418. Interestingly, *trk1*Δ *trk2*Δ mutants were resistant to G418 relative to wt and *hal4*Δ mutants. Hence, the degree to which KCl suppresses sensitivity is dependent on the particular drug in question (Table 2 and Fig. 3). Nevertheless, our findings clearly show that the exogenous elevation of external K^+^, Na^+^ or Rb^+^ ion concentrations suppresses the sensitivity of *S*. *pombe* of a large number of diverse drug classes.

Previous studies suggest that membrane polarization serves as a pleotropic drug resistance mechanism. Membrane potential in *S*. *pombe* is regulated by the antagonistic relationship between K^+^ import and proton export by Pma1 plasma membrane ATPase [8, 12, 13, 30]. The inability of *hal4* or *trk1 trk2* double mutants to effectively import K^+^ results in membrane hyperpolarization and increased sensitivity to metal cations and cationic drugs [8]. External addition of low KCl concentrations (50 mM) suppressed the sensitivity of these mutants to various cationic molecules [8]. Similarly, *S*. *cerevisiae* Pma1 mutants have hyperpolarized cell membranes and are resistant to hygromycin [13]. These findings suggest that membrane polarization influences sensitivity to cations. In our studies, external KCl concentrations of at least 150 mM were required to completely suppress drug sensitivity in wt *S*. *pombe* cells. Microscopic analyses using doxorubicin demonstrated that KCl prevents the import of the drug. Furthermore, *pzh1*Δ mutants are unable to effectively export K^+^ ions leading to membrane depolarization [12] and were partially resistant to G418 and phleomycin. We also demonstrated that Pzh1 and Sod2 respectively required for K^+^ and Na^+^ export were not required for the suppression of drug sensitivity by KCl. The *C*. *albicans* and *S*. *cerevisiae* homologues of *S*. *pombe* Pzh1, *CaPpz1* and *Ppz1* respectively, have similarly been linked to hygromycin B and spermine resistance [11, 39]. The partially conserved function of Pzh1 family proteins thus suggests a conserved functional role that influences drug resistance. Furthermore, Sty1 activity was required for tolerating exposure to KCl but not its effect on drug sensitivity. Our findings thus support the notion that membrane polarization confers pleiotropic drug resistance in *S*. *pombe* [8]. The differential sensitivity of bleomycin and phleomycin to external K^+^ concentrations compared to G418 and hygromycin suggests however, that membrane polarity alone cannot account for the suppressive effect of this ion on drug sensitivity. Mutants lacking the Trk1 and Trk2 K^+^ transporters must clearly still be able to import this ion. It has been proposed that amino acid permeases and glucose transporters may facilitate K^+^ import in the absence of Trk1 and Trk2 [40, 41]. Furthermore, the existence of a membrane potential- and voltage- sensitive ATPase alternative K^+^ importer in *S*. *pombe* has been proposed [42]. The L-carnitine transporter Agp2 in *S*. *cerevisiae* has been shown to mediate bleomycin uptake [43, 44]. Exposure to high KCl concentrations may alter the substrate specificity or uptake kinetics of these transporters. This may also account for the differential effect of KCl on sensitivity to bleomycin, hygromycin and G418 observed by us and others [8]. In *S*. *cerevisiae*, the drug:H^+^ transporters Qdr2 and Qdr3 facilitate resistance to bleomycin, cisplatin, spermine and other toxic compounds [45, 46]. In addition, Qdr2 and Qdr3 also play a role in regulating K^+^ concentrations in cells by facilitating the import of the ion [47]. These drug:H^+^ transporters may thus play a role in mediating the KCl- induced suppression of drug sensitivity. Our unexpected finding that co-deletion of *trk1* ^*+*^ and *trk2* ^*+*^ conferred resistance to G418 suggests that the Trk1 and Trk2 transporters may be involved in the uptake of this drug (Fig. 3).

We also examined the effect of high external KCl concentrations on the sensitivity of *S*. *pombe* to the ionophores nigericin and valinomycin. Nigericin is an H^+^/ K^+^ exchanger and induces cell death in part by causing the leakage of the latter ion from the cell [42, 48]. In our studies, high external KCl concentrations inhibited sensitivity to nigericin in *S*. *pombe*. Although previous reports demonstrated that high external K^+^ and Na^+^ concentrations suppress nigericin activity [48], we have now demonstrated that NH_4_Cl exerts a similar effect. Nigericin is anionic at physiological pH, suggesting that the protective effect of KCl and NH_4_Cl was not due to their effect on membrane potential. The precise mechanism whereby KCl and NH_4_Cl suppress sensitivity to nigericin remains unclear. One likely possibility is that they override the physiological effects of ion efflux induced by this ionophore [48]. In contrast to nigericin, valinomycin induces K^+^ influx in *S*. *pombe* [48] and did not affect viability in our assays. Strikingly, co-exposure to valinomycin and high KCl concentrations did result in a significant loss of viability. Co- exposure to NH_4_Cl and valinomycin affected viability to a far lesser degree. It is possible that in the presence of valinomycin, high external KCl concentrations result in the accumulation of toxic levels of K^+^ ions. Mutants lacking Pzh1 are unable to export K^+^ ions and are thus similarly sensitive to high KCl concentrations [12]. KCl also influenced the sensitivity of *S*. *pombe* to the polyene antibiotics amphotericin B and nystatin. At lower external KCl concentrations (0.06 M), sensitivity to amphotericin B was suppressed. In contrast, we did not observe this effect when the external KCl concentration was raised to 0.6 M (S4A Fig.). At this concentration, co-exposure to KCl also leads to a loss of viability in the presence of otherwise non-lethal concentrations of nystatin. The polyene antibiotics bind ergosterol in the fungal cell membrane, causing pore formation and ion leakage from the cell [21]. Low external KCl concentrations may compensate for the drug-induced loss of intracellular ions, while higher concentrations are toxic as a consequence of membrane disruption. KCl did not suppress sensitivity to clomitrazole, which disrupts the cell membrane by inhibiting ergosterol synthesis. A previous study in *S*. *cerevisiae* demonstrated that co-exposure 150 mM KCl, but not 300 mM sorbitol increased sensitivity to fluconazole [22]. Future studies will investigate the role of K^+^ ion homeostasis in modulating the sensitivity of *S*. *pombe* to azoles. It remains possible that lower concentrations of KCl or higher concentrations of sorbitol can suppress sensitivity to azoles. Both KCl and sorbitol activate the MAPK- regulated stress response pathway, which has been shown to mediate resistance to azoles in yeast (reviewed in [21]). Inhibition of the heat shock protein Hsp90, a molecular chaperone and downstream target of the MAPK pathway has been shown to enhance sensitivity to fluconazole in both *S*. *cerevisiae* and *C*. *albicans* [21, 49, 50]. KCl and sorbitol may thus influence sensitivity to azoles via activation of the MAPK- regulated stress response pathway. Additionally, low doses of KCl may counteract the disruptive effects of azoles on cation homeostasis [51]. Sensitivity to the echinocandin caspofungin, another disruptor of fungal cell membranes, was suppressed by KCl and sorbitol. Unlike the other antifungal drugs tested by us, caspofungin is water soluble, possibly explaining the protective effect of KCl. Clinically, the emergence of resistance to echinocandin antifungals has been linked to the MAPK- regulated stress response pathway which plays a role in maintaining cell wall integrity [21, 49]. Both KCl and sorbitol activate the MAPK- regulated stress response pathway in *S*. *pombe*. Hence, activation of these pathways by either compound may contribute towards suppressing sensitivity to caspofungin.

Our studies clearly indicate that external K^+^ and other ion concentrations modulate the sensitivity of *S*. *pombe* to a diverse array of ions and drugs. These findings raise a number of interesting questions. *S*. *pombe* is clearly suited to tolerate relatively high concentrations of K^+^ ions in the surrounding medium. The evolutionary significance of this ability remains unclear. *S*. *pombe* is frequently found in environments with relatively high potassium concentrations e.g grapes and millet [14]. Furthermore, potassium concentrations are can be particularly high in desiccating environments [52]. *S*. *pombe* likely competes for limited nutrients with antibiotic-producing microorganisms [53]. It is tempting to imagine that high potassium concentrations may confer a competitive advantage to *S*. *pombe* by facilitating resistance to antibiotics. Resistance to antifungal agents remains an important issue clinically. Furthermore, the use of polyene antibiotics is associated with significant toxicity. Our findings and those of others [8], suggest that drugs which interfere with ion homeostasis could be used to modulate the sensitivity of fungal pathogens to fungicides. Importantly, we demonstrate that it is the K^+^ and other cations and not osmotic stress that suppress sensitivity to various drugs. Nonetheless, activation of the MAPK- regulated stress response pathway by KCl and osmotic stress may suppress sensitivity to some antifungal agents.

## Conclusions

We have demonstrated that high external concentrations of K^+^ and some other alkali ions significantly suppress the sensitivity of *S*. *pombe* to numerous antibiotic and cytotoxic compounds. The ability of KCl to suppress drug sensitivity was not limited to any particular drug class. Nevertheless, KCl was particularly effective at suppressing sensitivity to cationic compounds. Changes in membrane polarization are thus likely to underlie this effect. However, changes to the specificity and kinetics of membrane transporters may also be involved. Our findings suggest that potassium rich environments may allow *S*. *pombe* to compete more effectively with organisms that produce antimicrobial agents in its natural environment. Modulating potassium homeostasis in fungal pathogens may also provide a strategy to suppress their resistance to some antifungal agents.

## Supporting Information

S1 Fig Effect of KCl on drug sensitivity in *S*. *pombe*. **A**. Wt and *rad24*Δ mutant strains were incubated in the presence of 10 μg/ ml phleomycin alone or with the indicated concentrations of KCl. Equal cell numbers were plated on YES agar and incubated for 2–3 days at 30°C. **B- D.** Minimum inhibitory concentrations (MICs) for phleomycin, G418 and hygomycin B in wt *S*. *pombe* cells was determined by incubating cultures for 24 h in the presence of the indicated drug concentrations. **E.** *S*. *pombe* cells were incubated with 10 μg/ ml phleomycin, or phleomycin (10 mg/ ml) incubated in an equal volume of 0.6 M KCl for 1 h and then diluted to 10 μg/ ml. The cells were exposed for 4 h and then plated in equal numbers on YES agar. **F.** *rad24*Δ mutants were exposed to 10 μg/ ml phleomycin alone and with the indicated concentrations of KCl for 4 h. The cells were fixed in 70% ethanol, stained with DAPI and examined by fluorescence microscopy. Arrows indicate cells with mis-segregated chromosomes. **G.** Wt cells were treated with 10 μM latrunculin B (LatB) or 50 μg/ ml MBC for 4 h and 7 h respectively, fixed in ethanol and treated as in G.
(PPTX)

S2 Fig Effect of external ions on drug and metal sensitivity in *S*. *pombe*. **A.** Cells were exposed to 10 μg/ ml phleomycin ± the indicated concentrations of RbCl for 24 h. Equal numbers of cells were plated on YES agar. **B.** Cells were treated as in A with phleomycin ± the indicated concentrations of NH_4_Cl. **C- D.** Cells were treated as in A but with the indicated compounds. **E.** Relative growth of *S*. *pombe* cells in the presence of 10 μg/ ml phleomycin ± the indicated compounds for 24h. Data represent the means of 3 experiments ± S.E. **F.** *S*. *pombe* cells were exposed to 0.4% acetic acid for 24 h and treated as in A. **G.** Wild type cells were exposed to the indicated compounds for 24 h and treated as in A. **H.** Wild type *S*. *pombe* cells were incubated with 40 μg/ ml doxorubicin alone and with 0.6 M KCl or 1.2 M sorbitol for 2 h and examined by fluorescent microscopy.
(PPTX)

S3 Fig KCl suppresses drug sensitivity in *S*. *pombe* independently of Sty1, Sod2, Pzh1 and Hal4. **A.** Wt and *sty1*Δ strains were incubated with the indicated concentrations of G418 for 24 h. Equal cell numbers were plated on YES agar and incubated at 30°C for 2–3 days. **B.** Wt and *sod2*Δ strains were incubated for 4 h in the presence of 20 μg/ ml phleomycin ± 0.6 M KCl and then treated as in A. **C.** *pzh1*Δ mutants were incubated for 4 h in the presence of 10 μg/ ml phleomycin ± 0.3 M KCl and then treated as in A. **D.** *pzh1*Δ mutants from C were fixed in 70% ethanol, stained with DAPI and examined by florescence microscopy. **E.** Wild type, *hal4*Δ and *sod2*Δ strains were incubated with 0.004 M LiCl for 4 h and then treated as in A.
(PPTX)

S4 Fig Effect of KCl on the sensitivity of *S*. *pombe* to cell wall disrupting agents. **A.** *S*. *pombe* cells were exposed to 1.0 μg/ ml amphotericin B ± the indicated concentrations of KCl for 24 h. Equal cell numbers were plated on YES agar and incubated at 30°C for 2–3 days. **B.** Cells were exposed to 1.0 μg/ ml caspofungin alone or together with the indicated concentrations of sorbitol and treated as in A.
(PPTX)

S5 Fig Molecular structures of compounds investigated in this study. Figures were obtained from the supplier web site or www.wikipedia.com.
(PPTX)
