# Supplementary material for: Suppression of Sensitivity to Drugs and Antibiotics by High External Cation Concentrations in Fission Yeast
Source: PLoS One. 2015 Mar 20;10(3):e0119297. doi: 10.1371/journal.pone.0119297 (PMC4368599; doi:10.1371/journal.pone.0119297)
Supplement: S1 Fig — A. Wt and rad24Δ mutant strains were incubated in the presence of 10 μg/ ml phleomycin alone or with the indicated concentrations of KCl. Equal cell numbers were plated on YES agar and incubated for 2–3 days at 30°C. B- D. Minimum inhibitory concentrations (MICs) for phleomycin, G418 and hygomycin B in wt S. pombe cells was determined by incubating cultures for 24 h in the presence of the indicated drug concentrations. E. S. pombe cells were incubated with 10 μg/ ml phleomycin, or phleomycin (10 mg/ ml) incubated in an equal volume of 0.6 M KCl for 1 h and then diluted to 10 μg/ ml. The cells were exposed for 4 h and then plated in equal numbers on YES agar. F. rad24Δ mutants were exposed to 10 μg/ ml phleomycin alone and with the indicated concentrations of KCl for 4 h. The cells were fixed in 70% ethanol, stained with DAPI and examined by fluorescence microscopy. Arrows indicate cells with mis-segregated chromosomes. G. Wt cells were treated with 10 μM latrunculin B (LatB) or 50 μg/ ml MBC for 4 h and 7 h respectively, fixed in ethanol and treated as in G. (PPTX) [file pone.0119297.s001.pptx]

## Slide 1
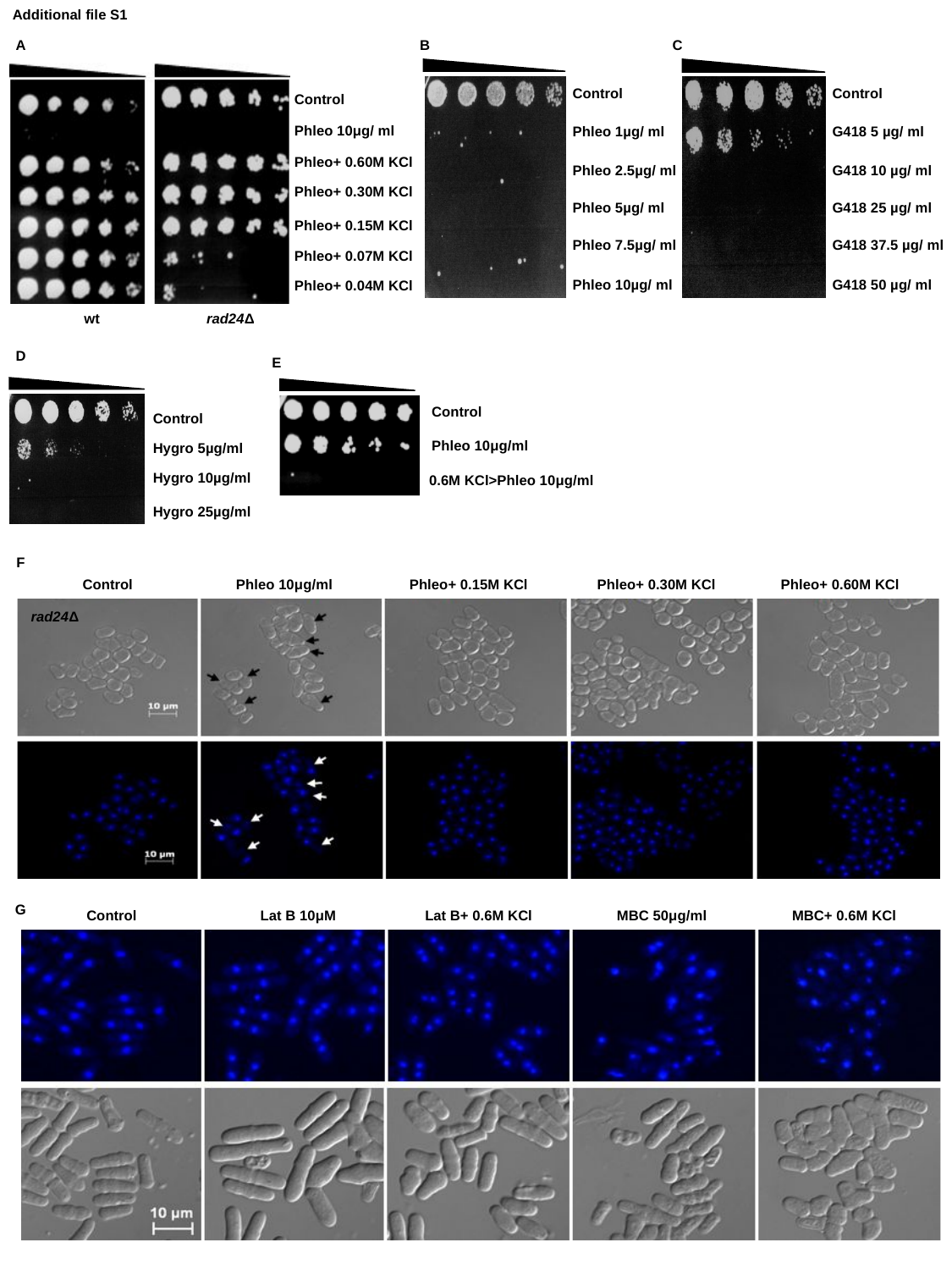

Additional file S1
A
B
C
Control
Control
Control
Phleo 10μg/ ml
Phleo 1µg/ ml
G418 5 µg/ ml
Phleo+ 0.60M KCl
Phleo 2.5µg/ ml
G418 10 µg/ ml
Phleo+ 0.30M KCl
Phleo 5µg/ ml
G418 25 µg/ ml
Phleo+ 0.15M KCl
Phleo 7.5µg/ ml
G418 37.5 µg/ ml
Phleo+ 0.07M KCl
Phleo 10µg/ ml
G418 50 µg/ ml
Phleo+ 0.04M KCl
wt
rad24Δ
D
E
Control
Control
Phleo 10μg/ml
Hygro 5µg/ml
Hygro 10µg/ml
0.6M KCl>Phleo 10μg/ml
Hygro 25µg/ml
F
Control
Phleo 10μg/ml
Phleo+ 0.15M KCl
Phleo+ 0.30M KCl
Phleo+ 0.60M KCl
rad24Δ
G
Control
Lat B 10μM
Lat B+ 0.6M KCl
MBC 50μg/ml
MBC+ 0.6M KCl
