# Supplementary material for: Suppression of Sensitivity to Drugs and Antibiotics by High External Cation Concentrations in Fission Yeast
Source: PLoS One. 2015 Mar 20;10(3):e0119297. doi: 10.1371/journal.pone.0119297 (PMC4368599; doi:10.1371/journal.pone.0119297)
Supplement: S2 Fig — A. Cells were exposed to 10 μg/ ml phleomycin ± the indicated concentrations of RbCl for 24 h. Equal numbers of cells were plated on YES agar. B. Cells were treated as in A with phleomycin ± the indicated concentrations of NH4Cl. C- D. Cells were treated as in A but with the indicated compounds. E. Relative growth of S. pombe cells in the presence of 10 μg/ ml phleomycin ± the indicated compounds for 24h. Data represent the means of 3 experiments ± S.E. F. S. pombe cells were exposed to 0.4% acetic acid for 24 h and treated as in A. G. Wild type cells were exposed to the indicated compounds for 24 h and treated as in A. H. Wild type S. pombe cells were incubated with 40 μg/ ml doxorubicin alone and with 0.6 M KCl or 1.2 M sorbitol for 2 h and examined by fluorescent microscopy. (PPTX) [file pone.0119297.s002.pptx]

## Slide 1
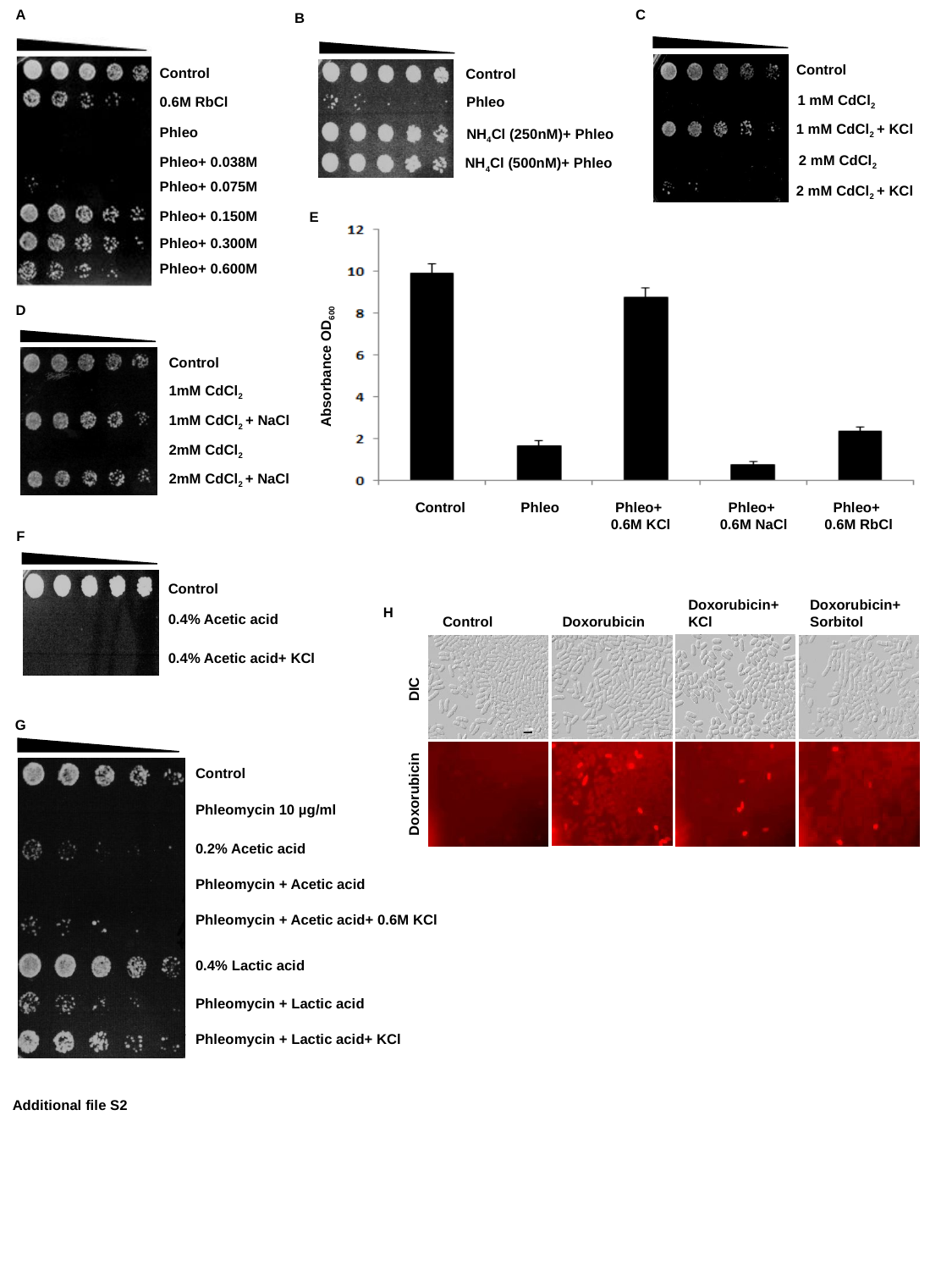

A
C
B
Control
Control
Control
1 mM CdCl2
0.6M RbCl
Phleo
1 mM CdCl2 + KCl
Phleo
NH4Cl (250nM)+ Phleo
2 mM CdCl2
Phleo+ 0.038M
NH4Cl (500nM)+ Phleo
Phleo+ 0.075M
2 mM CdCl2 + KCl
Phleo+ 0.150M
E
Phleo+ 0.300M
Phleo+ 0.600M
D
Control
Absorbance OD600
1mM CdCl2
1mM CdCl2 + NaCl
2mM CdCl2
2mM CdCl2 + NaCl
Control
Phleo
Phleo+
0.6M KCl
Phleo+
 0.6M NaCl
Phleo+
 0.6M RbCl
F
Control
Doxorubicin+ KCl
Doxorubicin+ Sorbitol
H
0.4% Acetic acid
Control
Doxorubicin
0.4% Acetic acid+ KCl
DIC
G
Control
Doxorubicin
Phleomycin 10 µg/ml
0.2% Acetic acid
Phleomycin + Acetic acid
Phleomycin + Acetic acid+ 0.6M KCl
0.4% Lactic acid
Phleomycin + Lactic acid
wt
Phleomycin + Lactic acid+ KCl
Additional file S2
