# Supplementary material for: Suppression of Sensitivity to Drugs and Antibiotics by High External Cation Concentrations in Fission Yeast
Source: PLoS One. 2015 Mar 20;10(3):e0119297. doi: 10.1371/journal.pone.0119297 (PMC4368599; doi:10.1371/journal.pone.0119297)
Supplement: S3 Fig — A. Wt and sty1Δ strains were incubated with the indicated concentrations of G418 for 24 h. Equal cell numbers were plated on YES agar and incubated at 30°C for 2–3 days. B. Wt and sod2Δ strains were incubated for 4 h in the presence of 20 μg/ ml phleomycin ± 0.6 M KCl and then treated as in A. C. pzh1Δ mutants were incubated for 4 h in the presence of 10 μg/ ml phleomycin ± 0.3 M KCl and then treated as in A. D. pzh1Δ mutants from C were fixed in 70% ethanol, stained with DAPI and examined by florescence microscopy. E. Wild type, hal4Δ and sod2Δ strains were incubated with 0.004 M LiCl for 4 h and then treated as in A. (PPTX) [file pone.0119297.s003.pptx]

## Slide 1
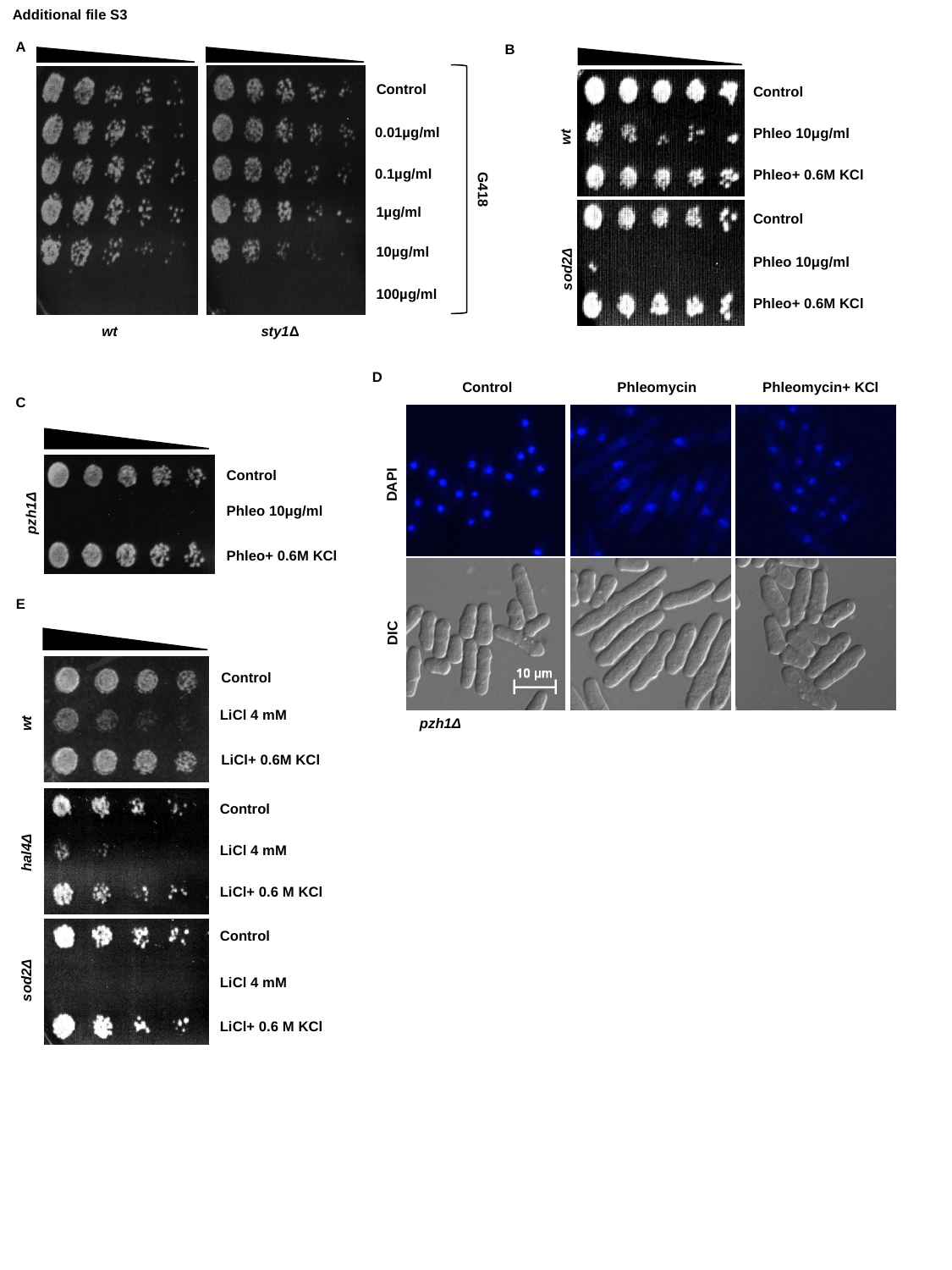

Additional file S3
A
B
Control
Control
0.01µg/ml
Phleo 10μg/ml
wt
0.1µg/ml
Phleo+ 0.6M KCl
G418
1µg/ml
Control
10µg/ml
Phleo 10μg/ml
sod2Δ
100µg/ml
Phleo+ 0.6M KCl
wt
sty1Δ
D
Control
Phleomycin
Phleomycin+ KCl
C
Control
DAPI
Phleo 10μg/ml
pzh1Δ
Phleo+ 0.6M KCl
E
DIC
Control
LiCl 4 mM
pzh1Δ
wt
LiCl+ 0.6M KCl
Control
LiCl 4 mM
hal4Δ
LiCl+ 0.6 M KCl
Control
sod2Δ
LiCl 4 mM
LiCl+ 0.6 M KCl
