# Supplementary material for: Suppression of Sensitivity to Drugs and Antibiotics by High External Cation Concentrations in Fission Yeast
Source: PLoS One. 2015 Mar 20;10(3):e0119297. doi: 10.1371/journal.pone.0119297 (PMC4368599; doi:10.1371/journal.pone.0119297)
Supplement: S4 Fig — A. S. pombe cells were exposed to 1.0 μg/ ml amphotericin B ± the indicated concentrations of KCl for 24 h. Equal cell numbers were plated on YES agar and incubated at 30°C for 2–3 days. B. Cells were exposed to 1.0 μg/ ml caspofungin alone or together with the indicated concentrations of sorbitol and treated as in A. (PPTX) [file pone.0119297.s004.pptx]

## Slide 1
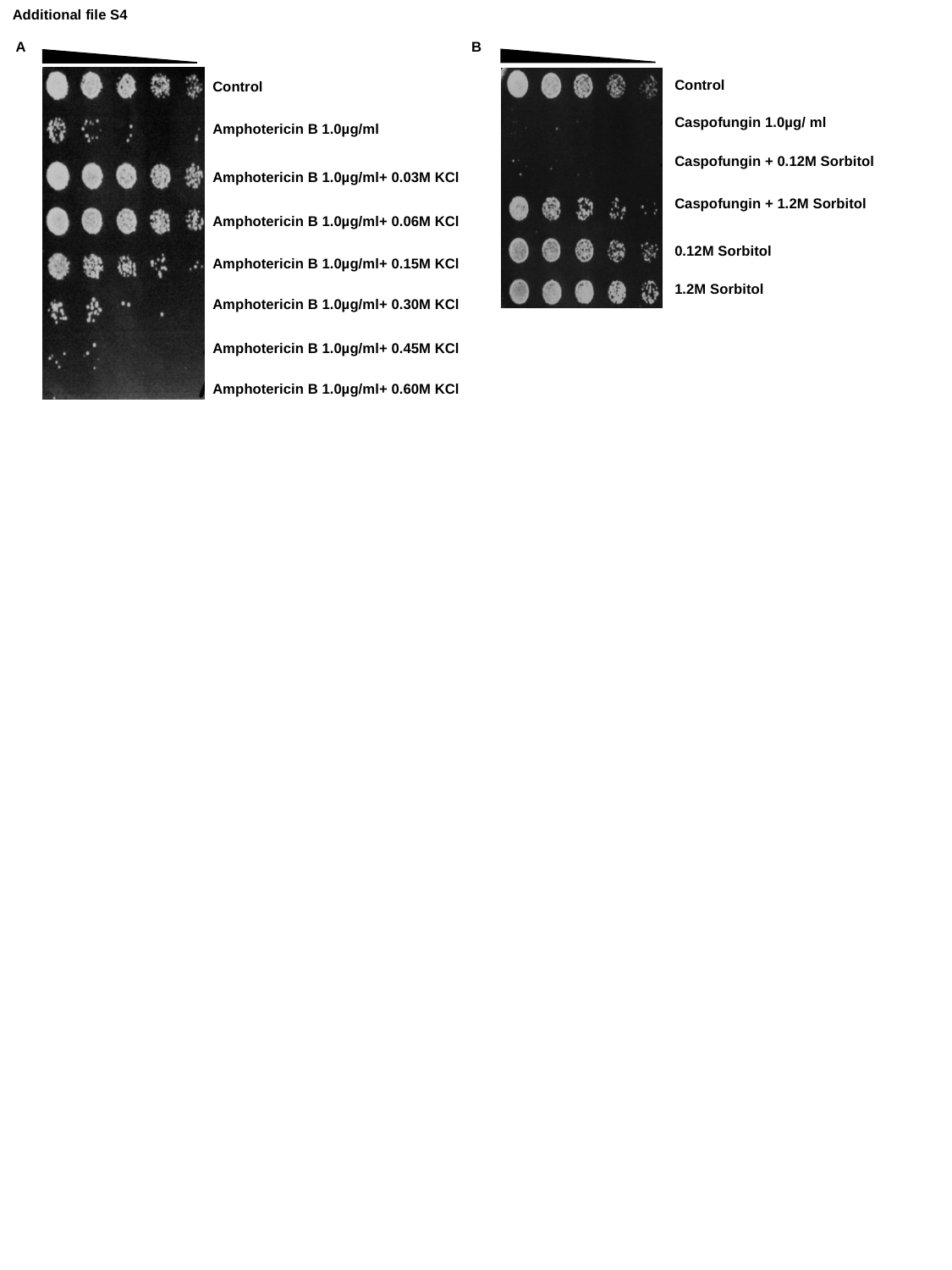

Additional file S4
A
B
Control
Control
Caspofungin 1.0µg/ ml
Amphotericin B 1.0µg/ml
Caspofungin + 0.12M Sorbitol
Amphotericin B 1.0µg/ml+ 0.03M KCl
Caspofungin + 1.2M Sorbitol
Amphotericin B 1.0µg/ml+ 0.06M KCl
0.12M Sorbitol
Amphotericin B 1.0µg/ml+ 0.15M KCl
1.2M Sorbitol
Amphotericin B 1.0µg/ml+ 0.30M KCl
Amphotericin B 1.0µg/ml+ 0.45M KCl
Amphotericin B 1.0µg/ml+ 0.60M KCl
