# Supplementary material for: Suppression of Sensitivity to Drugs and Antibiotics by High External Cation Concentrations in Fission Yeast
Source: PLoS One. 2015 Mar 20;10(3):e0119297. doi: 10.1371/journal.pone.0119297 (PMC4368599; doi:10.1371/journal.pone.0119297)
Supplement: S5 Fig — Figures were obtained from the supplier web site or www.wikipedia.com. (PPTX) [file pone.0119297.s005.pptx]

## Slide 1
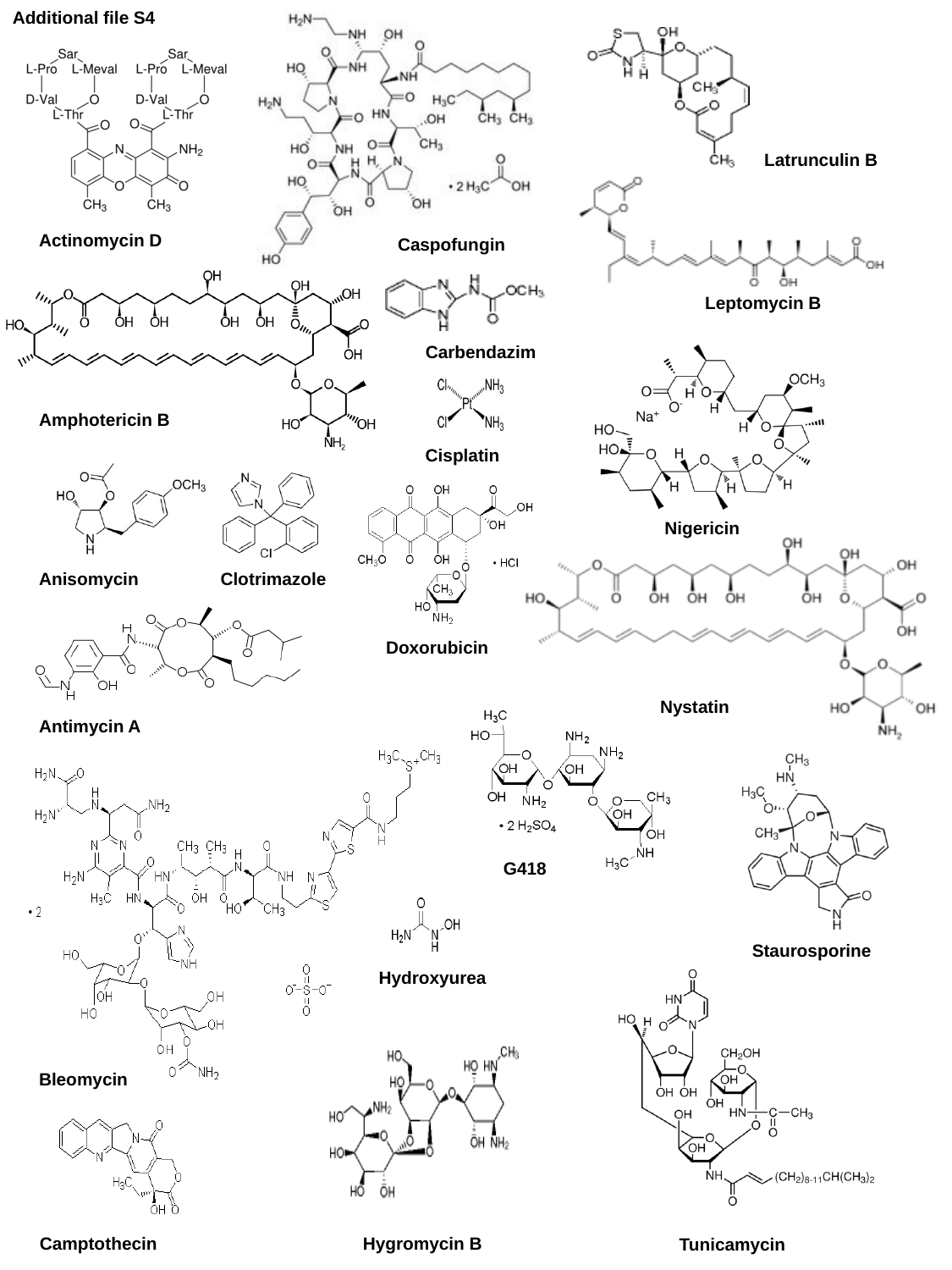

Additional file S4
Latrunculin B
Actinomycin D
Caspofungin
Leptomycin B
Carbendazim
Amphotericin B
Cisplatin
Nigericin
Anisomycin
Clotrimazole
Doxorubicin
Nystatin
Antimycin A
G418
Staurosporine
Hydroxyurea
Bleomycin
Camptothecin
Hygromycin B
Tunicamycin
